# Angiostrongyliasis in the Americas

**DOI:** 10.3201/eid1506.071708

**Published:** 2009-06

**Authors:** Alberto Juan Dorta-Contreras, María Esther Magraner-Tarrau, Eduardo Sánchez-Zulueta

**Affiliations:** Facultad de Ciencias Médicas “Dr. Miguel Enríquez,” Ciudad Habana, Cuba

**Keywords:** Angiostrongylus cantonensis, intrathecal synthesis, parasites, Americas, letter

**To the Editor**: We read with special interest the article by Hochberg et al. about angiostrongyliasis in Hawaii (*1*). *Angiostrongylus cantonensis* meningitis in the Americas was reported by Aguiar et al. in Cuba in 1981 (*2*), and we have studied this zoonosis during the ensuing 25 years. We agree with the authors about the difficulty in obtaining a specific immunoassay for detection of antibodies to *A. cantonensis* antigens. In Cuba, as in Hawaii, no other cause of eosinophilic meningitis was identified.

To improve accuracy of the diagnosis we investigated immunoglobulin (Ig) E intrathecal synthesis during the first diagnostic lumbar puncture. We also confirmed this synthesis as either a 2-class response (IgG + IgA) or a 3-class response (IgG + IgA + IgM) that appeared 8 days later in cerebrospinal fluid (*3*).

Since 1991, our records show that the major incidence of the disease is during the second quarter of the year. We detected 32% of the cases during the rainy season when rats come into houses in rural and semirural areas and snails and slugs appear more often in gardens and yards where children play. Ethnicity data show that 52% of those affected were Caucasian and 32% were African. The median interval from onset of symptoms to lumbar puncture was 1–3 days. Although no children died, 6 (23%) of 26 adult patients died. The clinical signs and symptoms of the Cuban patients are similar to those in Hawaii (*4*,*5*). We congratulate the authors for systematically determining incidence rates of *A. cantonensis* meningoencephalitis, a severe but preventable infection.
